# Laparoscopic Wedge Resection of a 6 cm Gastrointestinal Stromal Tumor (GIST) Arising From the Lesser Curvature: A Case Report From Jordan

**DOI:** 10.7759/cureus.98148

**Published:** 2025-11-30

**Authors:** Ramadan Hassanat, Mohammad Alhroot, Qutaiba Qatawneh, Eyad Rawashdeh, Alhareth Qatawneh, Rami Al-Omoor, Mutaz Haddadin, Mohammad Al-huniti, Omar Abu Zeitoun, Ghidaa Maswadah

**Affiliations:** 1 Department of General Surgery, Jordanian Royal Medical Services, Amman, JOR; 2 Department of Radiology, Jordanian Royal Medical Services, Amman, JOR; 3 Department of Anesthesiology, Jordanian Royal Medical Services, Amman, JOR

**Keywords:** case report, gastrointestinal stromal tumor, jordan, laparoscopy, wedge resection

## Abstract

Laparoscopic wedge resection of gastrointestinal stromal tumors (GISTs) is the preferred approach for small-sized tumors. Our case study aims to clarify the feasibility of laparoscopic resection of a 6 cm GIST from the lesser curvature in elderly patients, thereby avoiding major gastrectomy.

We present a 75-year-old female patient, diabetic and hypertensive, from Amman, Jordan, who had been suffering from epigastric pain and vomiting for a duration of three months. The computed tomography (CT) scan and endoscopy revealed a 6 cm gastric mass arising from the lesser curvature, 4 cm away from the gastroesophageal junction (GEJ), confirmed by endoscopic ultrasound-guided biopsy as a GIST. The patient was referred to a hematologist for possible mass regression with neoadjuvant imatinib. She was then returned to the surgical team after developing drug intolerance, which exacerbated vomiting. The decision was made for laparoscopic resection based on her clinical scenario, and after full preoperative preparation, the procedure was performed in June 2025 at King Hussein Medical Center.

The patient underwent full laboratory and radiological investigations before surgery. She also underwent an endoscopy for mass localization. The procedure was performed successfully through a laparoscopic approach via wedge resection using an endo gastrointestinal anastomosis (GIA) stapler over a 40 F (French) Bougie, followed by a negative methylene blue test. After surgery, the patient was monitored in the general ward and was found to be well. A Gastrografin study was performed to assess the gastric anatomy after resection and to rule out leakage. The patient was discharged on the first postoperative day after tolerating oral intake and was followed up as an outpatient for three months without significant complaints.

## Introduction

Gastrointestinal stromal tumors (GISTs) are the most common mesenchymal neoplasms of the gastrointestinal system; they arise from the cells of Cajal in the mesenchymal smooth muscle layer. Most tumors express mutations in KIT CD117 or platelet-derived growth factor receptor alpha (PDGFRA) genes [1]. More than 60% of tumors arise from the stomach, mainly along the greater curvature and the body, and are less commonly found on the lesser curvature, where surgery can be more challenging due to proximity to the gastroesophageal junction (GEJ) and the vagus nerve [1]. In contrast, GISTs arising on the lesser curvature are uncommon and pose specific surgical challenges due to the limited working space, proximity to major vascular structures, and risk of compromising the GEJ [2].

The malignant behavior of the disease is variable according to the site, size, and mitotic index. Usually, the good prognostic factors are gastric GISTs, tumor size less than 2 cm, and a low mitotic index [2]. These prognostic factors strongly affect surgical planning for tumors located on the lesser curvature, making achieving negative margins without excessive gastric resection difficult.

Usually, the tumor is discovered incidentally, but sometimes it presents with abdominal pain and vomiting, especially if it is near the GEJ [3]. The diagnosis is confirmed by biopsy through endoscopy or ultrasonography-guided fine-needle aspiration (FNA) [2]. Routine upper endoscopy (gastroscopy) often fails to obtain diagnostic tissue from suspected GISTs because these tumors arise in the submucosal or extraluminal layers, beyond the reach of standard mucosal biopsies. Endoscopic ultrasound (EUS) is a separate procedure that provides better visualization and enables tissue sampling through EUS-guided FNA or fine-needle biopsy (FNB), which is the preferred method for diagnosing GISTs [2]. CT scans and magnetic resonance imaging (MRI) can also help in the diagnosis and localization of the tumor [3]. Accurate preoperative localization is particularly crucial for the lesser-curvature GISTs, where even small deviations in tumor position influence the feasibility of a stomach-preserving wedge resection [3].

Surgical resection with free margins is the gold standard treatment option [4]. According to the European Society for Medical Oncology (ESMO), laparoscopic resection is the best option for tumors less than 2 cm and can be feasible for larger tumors with challenges [3]. However, tumors on the lesser curvature often need gastrectomy due to the strict mobility of the gastric wall and the reduced space to the GEJ, deeming wedge resection technically demanding and less commonly reported [2].

Tyrosine kinase inhibitors, imatinib (Gleevec), can be helpful in the case of large GISTs or with metastases and can also be used to shrink the tumor size before surgery or to decrease the recurrence rate after resection [3].

Only a limited number of reports describe successful stomach laparoscopic resection of lesser-curvature GISTs, especially in elderly patients, where reducing trauma is essential. Therefore, this study aims to highlight the feasibility of the laparoscopic wedge resection of gastric GIST located on the lesser curvature, as a stomach-preserving alternative to major gastrectomy, particularly in elderly patients.

## Case presentation

A 75-year-old, diabetic and hypertensive, female patient was referred from the Emergency Department of King Hussein Medical Center to the General Surgery Clinic. She complained of abdominal pain, stabbing in nature, radiating to the back, and associated with occasional nonprojectile vomiting of gastric contents without hematemesis. There was no recent weight loss or anorexia, and the condition was not associated with a change in bowel habits. Based on these findings, the surgical team planned a laparoscopic wedge resection, which was performed in June 2025 at King Hussein Medical Center.

**Figure 1:**
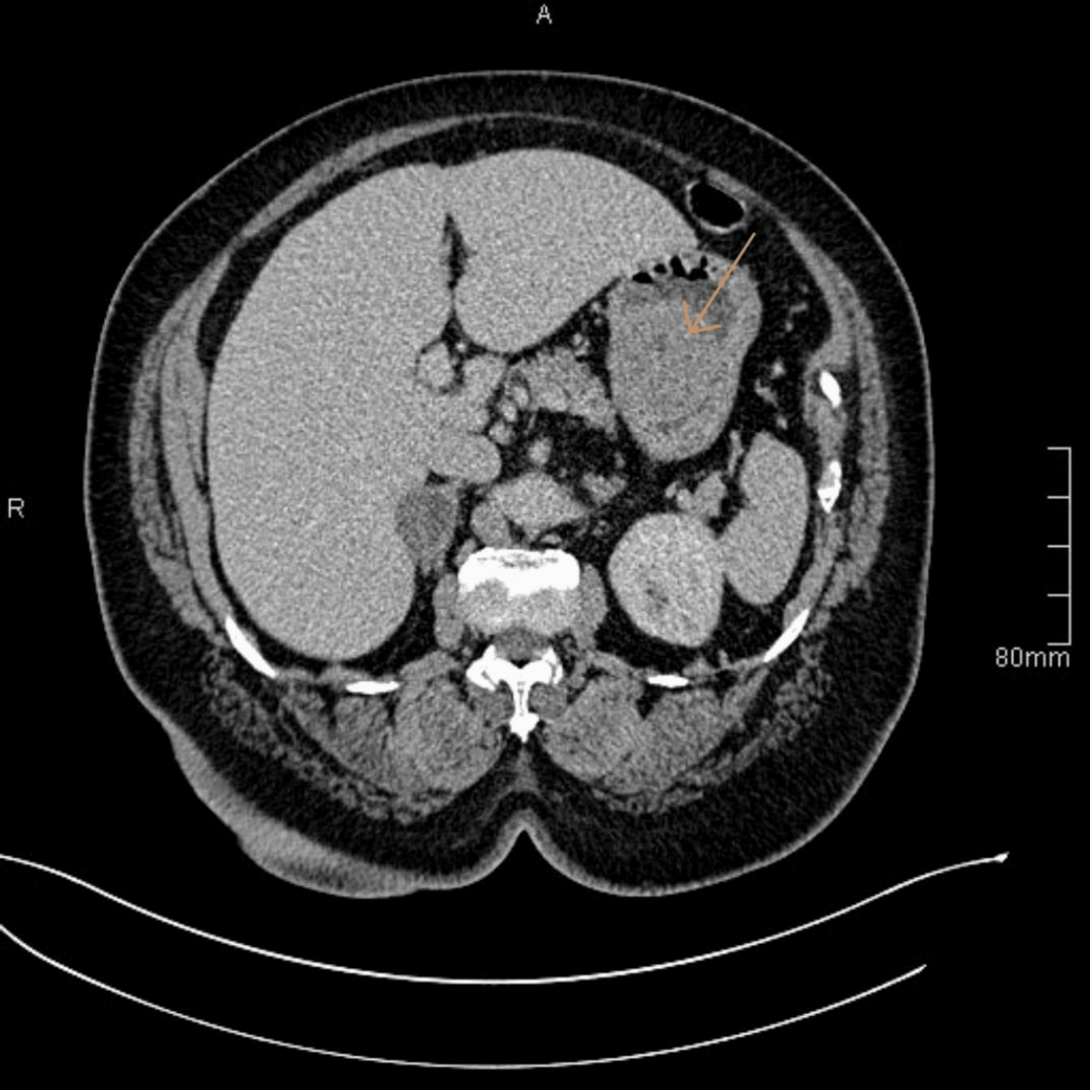
A CT of the abdomen shows a gastric mass on the lesser curvature, rounded without invasion or lymphadenopathy, measuring about 5 x 6 cm CT: computed tomography

Initial lab tests, complete blood count, kidney function test, and liver function test, were normal. CT scan showed a 5-6 cm well-circumscribed gastric mass, the mass was 4 cm away from the GEJ, located medially in the lesser curvature, with no signs of metastasis or lymphadenopathy (Table 1, Figure 1). 

**Table 1:** Complete blood count (CBC) WBC: white blood cell count; RBC: red blood cell count; HB: hemoglobin; HCT: hematocrit; MCV: mean corpuscular volume; MCH: mean corpuscular hemoglobin; MCHC: mean corpuscular hemoglobin concentration; PLT: platelet count; RDW-CV: red cell distribution width (coefficient of variation); MPV: mean platelet volume; NEUTROPHILS%: neutrophils percentage; NEUTROPHILS#: neutrophils absolute count; LYMPHOCYTES%: lymphocytes percentage; LYMPHOCYTES#: lymphocytes absolute count

Test	Result	Flag	Units	Reference range
WBC	5.745		10³/µL	3.4-9.6
RBC	4.49	-	10⁶/µL	3.92-5.13
HB	11.27	L	g/dL	11.6-15
HCT	38.15	-	%	35.6-44.9
MCV	85.01	-	fL	78.2-97.9
MCH	25.11	L	pg	26-34
MCHC	29.64	L	g/dL	32-36
PLT	268.5	-	10³/µL	157-371
RDW-CV	15.0	-	%	12.2-16.1
MPV	8.054	-	fL	-
NEUTROPHILS%	80.31	-	%	-
NEUTROPHILS#	4.614	-	10³/µL	1.66-6.45
LYMPHOCYTES%	17.75	-	%	-
LYMPHOCYTES#	1.020	-	10³/µL	0.95-3.07

The recommended plan from the multidisciplinary team meeting was to proceed with gastric-preserving laparoscopic wedge resection to avoid major gastrectomy and minimize the surgical risk in an elderly patient with several comorbidities [1,2].

Preoperative preparations

The patient was scheduled for elective surgery and referred to an endocrinologist for consultation regarding her diabetes mellitus (DM); her glycated hemoglobin (HbA1c) was 7%, with controlled random blood sugar. Cardiologists and pulmonologists gave the clearance for surgery. On the night of surgery, the patient underwent an endoscopy for tumor localization (Table 2).

**Table 2:** Serum chemistry ALT: alanine aminotransferase; AST: aspartate aminotransferase; bilirubin, total: total bilirubin; BUN: blood urea nitrogen; glucose, serum: blood glucose (serum); protein, total: total protein

Test	Result	Flag	Units	Reference range
ALT	14		U/L	≤41
AST	17	-	U/L	≤37
Bilirubin, total	0.8	-	mg/dL	1-1.2
Creatinine	0.93	-	mg/dL	0.5-1.2
BUN	20	-	mg/dL	6-20
Glucose, serum	104	-	mg/dL	70-110
Sodium	143	-	mEq/L	135-153
Potassium	4.3	-	mEq/L	3.5-5.5
Calcium	8.6	-	mg/dL	8.4-10.5
Uric acid	6.5	H	mg/dL	2.7-6.1
Protein, total	74	-	g/L	60-87

**Figure 2:**
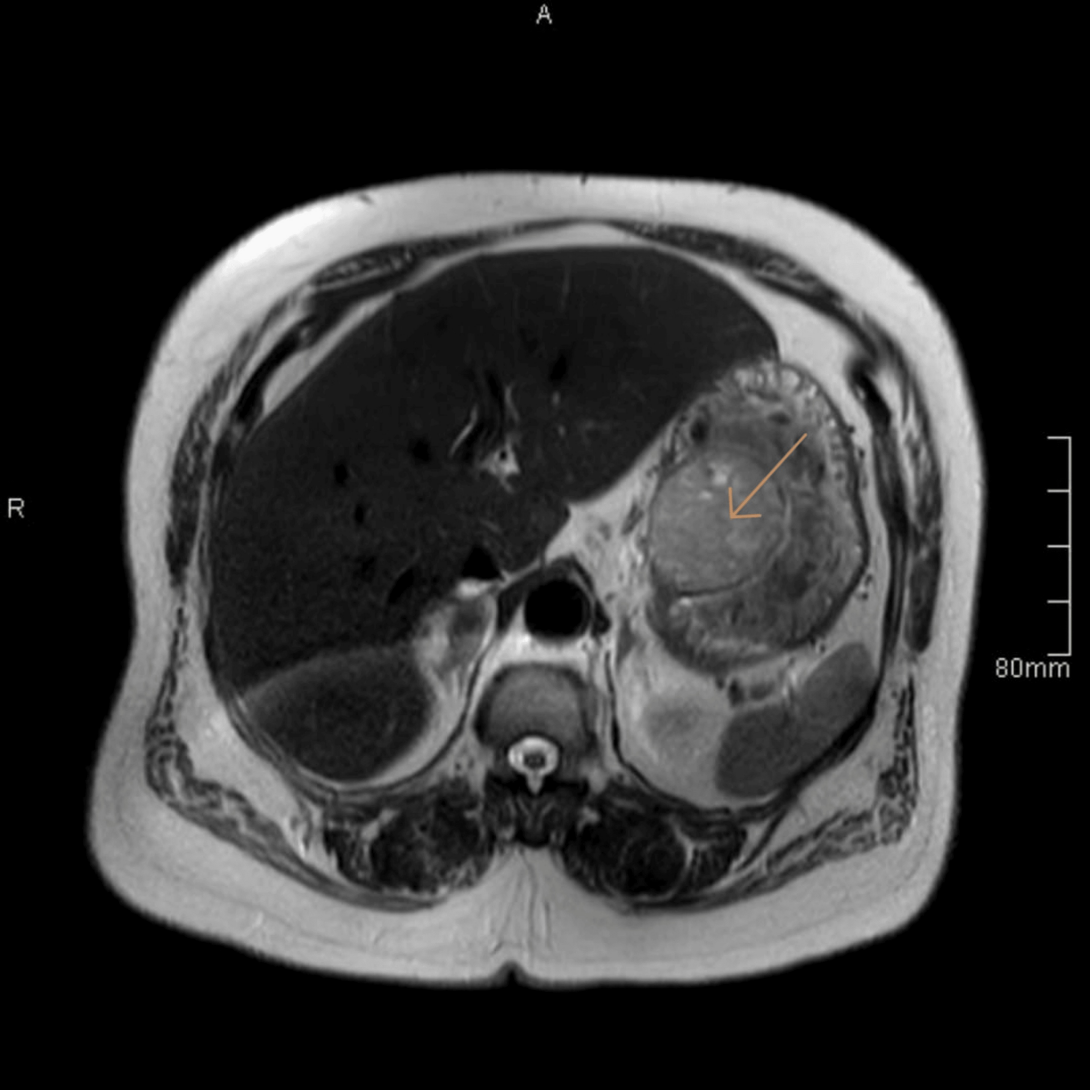
A well-circumscribed, exophytic soft tissue mass lesion arising from the inner surface of posteriomedial gastric wall The lesion shows heterogeneous signal intensity on T2-weighted MRI, with central areas of necrosis or cystic degeneration, and shows no definite invasion or extra gastric extension

**Figure 3:**
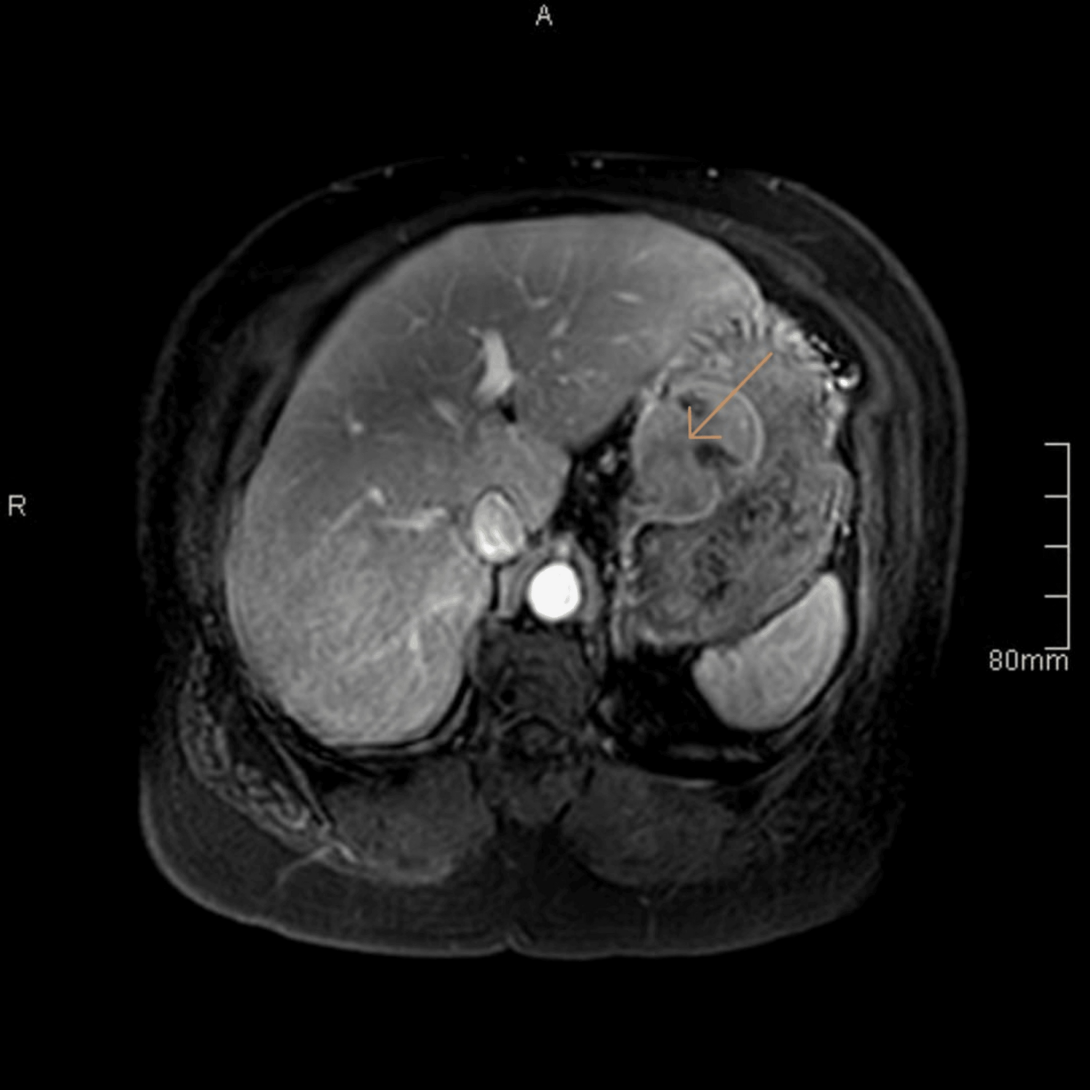
Contrast-enhanced T1-weighted MRI (post-contrast images) demonstrates heterogeneous enhancement and shows no definite invasion or extra gastric extension

Endoscopy showed a smooth submucosal bulge, overlaid with a thin and intact mucosa, representing typical features of GIST. For further work-up, abdominal MRI, T1, and T2-weighted MRI were performed (Figures 2, 3). The histopathological diagnosis is confirmed by EUS-guided biopsy. The patient was referred to the oncologist for the possibility of neoadjuvant imatinib to shrink the mass. The oncology team advised proceeding with surgery after drug intolerance (she suffered from an exacerbation of vomiting). Together, the presence of a defined submucosal mass on CT, a smooth bulge on endoscopy, and an exophytic lesion on MRI strongly indicated a gastric GIST.

The surgical procedure

After confirming the diagnosis and assessing surgical risk, the patient proceeded to laparoscopic wedge resection. In the morning on the day of the procedure, the patient was fasting for 12 hours and had prepared two units of packed red blood cells (PRBCs). After general anesthesia with endotracheal intubation, scrubbing, and skin draping, the patient was placed in the French position with reverse Trendelenburg, and pneumoperitoneum was created by the insertion of a Veress needle in Palmer’s point [3].

The procedure began with the dissection of the lesser sac using a bipolar ligasure; the right and left crura were exposed. The second step was the insertion of a Bougie size 40 F peroral tube until reaching the pylorus.

The resection was performed using 5 units of the endo-gastrointestinal anastomosis (GIA) stapler 60 mm. Lastly, multiple endoclips were applied at the stapler edge to secure hemostasis. The resected mass was extracted from the abdominal cavity via Endo-Bag to avoid spillage. The closure was performed after a methylene blue test and easy Bougie mobilization through the stomach [3].

Postoperative

On the 1st day after surgery, the patient was doing clinically well with normal vital signs and no significant changes in packed cell volume (PCV). The patient was sent to the radiology department for an oral Gastrografin test to rule out leakage or stenosis, and the result was normal. Then, she started a clear liquid diet and was discharged after tolerating oral intake, with extended antithrombotic injections for 14 days.

**Figure 4:**
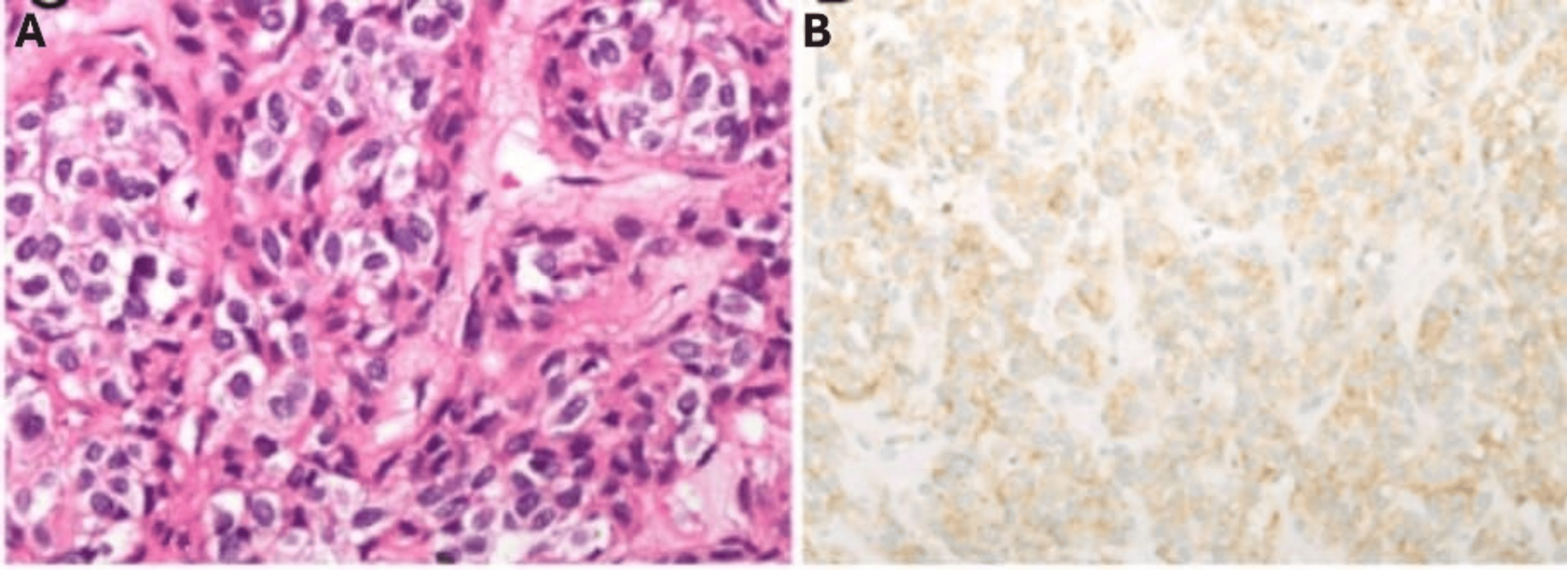
Microscopic image of GIST. (A) Image for epithelioid tumor cells, stained by H&E, under a high power field (x200). (B) A positive cytoplasmic signal of CD 117, appeared in the tumor cell (×200) GIST: gastrointestinal stromal tumor; H&E: hematoxylin and eosin

The 1st outpatient clinic was on the 14th day after surgery, the patient's laboratory tests were normal, and a dietitian consultation was conducted. Histopathological report revealed GIST with complete resection. The patient was referred to the hematologist to start imatinib as adjuvant therapy (Table 3, Figure 4). This case displays how multiple symptom assessments, targeted imaging, and EUS-guided biopsy can reliably identify lesser-curvature GISTs and guide stomach-preserving surgical management.

**Table 3:** Histopathology report GIST: gastrointestinal stromal tumor; pTNM: pathological tumor-node-metastasis

Parameter	Findings
Tumor site	Stomach
Histological type	GIST, spindle cell type
Size	6 cm (greatest dimension)
Grade	G1 (low grade)
Necrosis	Present (20%)
Margins	All negative; closest 1 mm
Risk category	Low (3.6%)
pTNM (AJCC 8th)	pT3
Final diagnosis	Gastric GIST, low grade, R0 resection (6 cm, low-risk)

**Figure 5:**
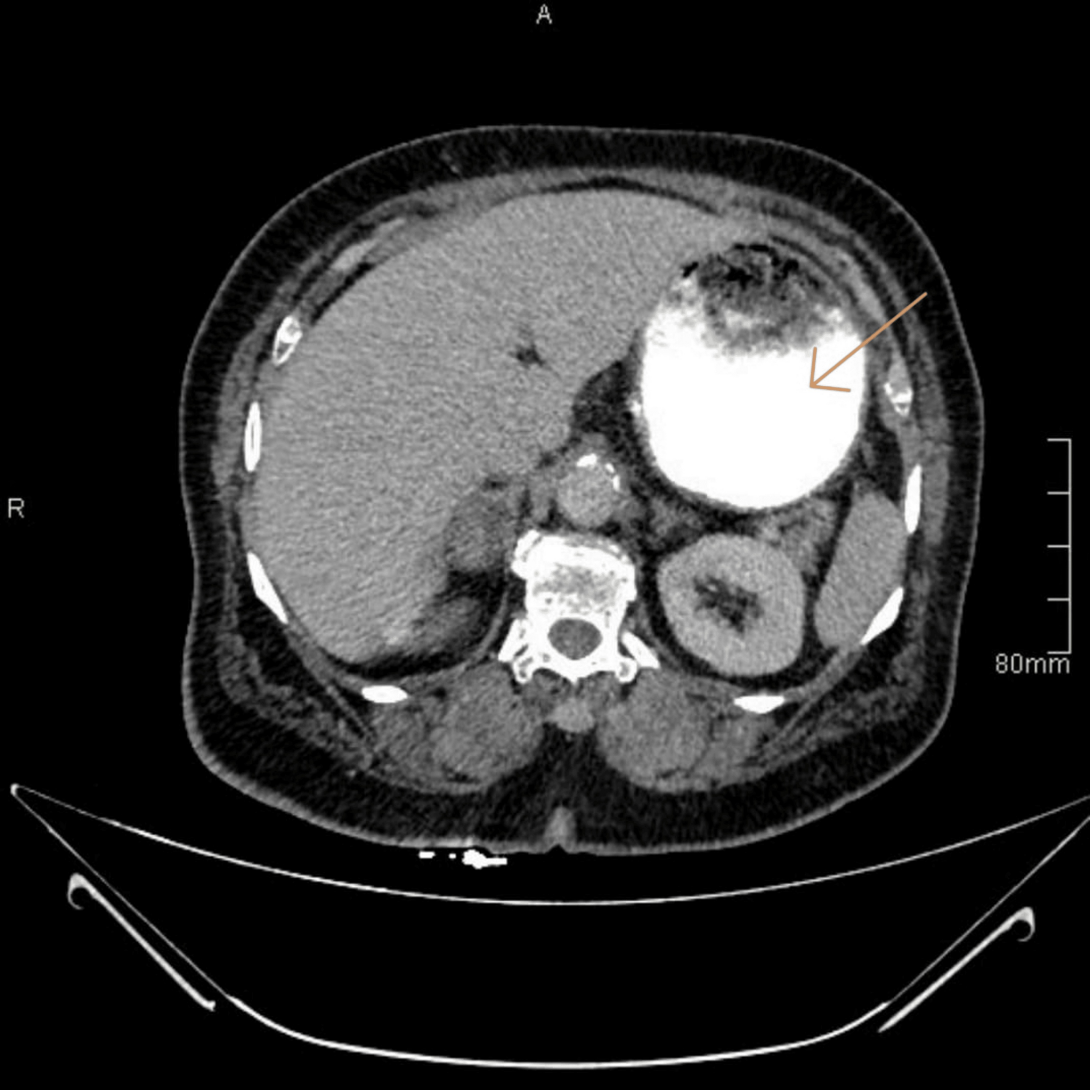
After surgery, abdominal CT, coronal section, with IV and oral contrast, shows no masses with stomach full of contrast; the oral contrast swallowed immediately before scanning

After 30 days from the date of surgery, the patient came back to our clinic with no complaints, a clean wound, and tolerance of oral intake. And finally, the last visit to the oncologist revealed complete recovery without remission or complications. A contrasted abdominal CT (with IV and oral contrast) was performed and showed no leakage or stenosis (Figure 5).

## Discussion

In the past, open resections of gastric GISTs with or without partial gastrectomy were the best modality; nowadays, in the era of laparoscopic surgery, the optimal choice for many operations has become minimally invasive surgery. Gastric GISTs are variable in size, site, and mitotic index [3].

According to the ESMO, laparoscopic resection is the best option for tumors less than 2 cm and may be feasible for larger tumors with challenges [3]. In addition, larger tumors can be resected laparoscopically with only a wedge resection to preserve the stomach or partial gastrectomy in case of tumor proximity to the GEJ or incisura [4]. In the literature review, there are many studies that describe the minimally invasive gastric-preserving wedge resection even for 5 cm or more gastric GIST [4-12]. Furthermore, Choi et al. described a case series study of 51 cases with variable tumor sites and sizes, which underwent laparoscopic resection with minimal comorbidity and good outcomes, especially for elderly patients. Minimal surgical resection with organ preservation is required [13].

In our presented case, the tumor was 6 cm, and it was 3 cm away from GEJ on the lesser curvature; the laparoscopic approach was challenging but feasible; we took into consideration the age of the patient. Geriatric patients need as minimal resection as possible to decrease the risk of complications. At the same time, the resection was complete with R0 in all tumor edges.

The lesser-curvature tumor requires an expert surgeon due to its proximity to the GEJ and vagus nerve. The goal is to achieve complete resection with minimal comorbidity, particularly in elderly patients. Yunus Donder, a Turkish surgeon, reported that tumors located along the lesser curvature can be resected safely without a gastric emptying problem. In his retrospective study of 30 patients, seven had tumors on the lesser curvature; three of these underwent open resection (tumor size was more than 10 cm), while the remaining four patients underwent successful laparoscopic wedge resection [14]. However, when the tumor is located medially or arises from the lesser curvature, delayed gastric emptying is a concerning complication. This has been reported in both open and laparoscopic approaches, as described by Waseda et al. [15].

On the other hand, some authors recommend open surgical resection for lesser-curvature tumors. Ceccarelli et al. advised that tumors close to the lesser curvature should be managed by a tailored open or hybrid surgical technique [16]. Similarly, Kong in 2013 concluded that the treatment of GIST near the GEJ is challenging and complex, and it carries a higher risk for gastric deformity with delayed gastric emptying [17].

One of the largest studies on gastric GIST was conducted at Nanfang Hospital, China, by Liao et al. The study included 204 cases over 10 years. The tumors were categorized according to the location (favorable vs. unfavorable sites), and the authors noted that the survival rates were similar between groups; the authors emphasized that laparoscopic resection in unfavorable sites should be performed only by trained and skilled surgeons. They conclude that all gastric GISTs, regardless of size, are potentially feasible for laparoscopic resection [18].

## Conclusions

In selected patients, laparoscopic stomach-preserving wedge resection of GIST located on the lesser curvature can be feasible, provided that careful technique is used to avoid gastric stenosis and minimize the morbidities associated with more extensive gastric resection. Further clinical trials and broader comparative studies are required to confirm its applicability.
